# An Integrative Human Pan-Cancer Analysis of Cyclin-Dependent Kinase 1 (CDK1)

**DOI:** 10.3390/cancers14112658

**Published:** 2022-05-27

**Authors:** Xuanyou Liu, Hao Wu, Zhenguo Liu

**Affiliations:** 1 Department of Medical Pharmacology and Physiology, University of Missouri School of Medicine, Columbia, MO 65212, USA; liuxua@health.missouri.edu; 2 Center for Precision Medicine and Division of Cardiovascular Medicine, Department of Medicine, University of Missouri School of Medicine, Columbia, MO 65212, USA; hw5mg@missouri.edu

**Keywords:** CDK1, pan-cancer, tumor, immune infiltration, prognosis, enrichment analysis

## Abstract

**Simple Summary:**

Cyclin-dependent kinase 1 (CDK1), one of the key regulators of the G2/M checkpoint, is expressed in many cells and plays an important role in cell cycle control. However, CDK1 expression is substantially increased in many tumors of diverse origins and is associated with tumorigenesis. Targeting CDK1 shows promising results for several tumors. However, a systematic and integrative analysis of CDK1 in cancer has not been conducted. The present study aims to use pan-cancer analysis to investigate the relationship, similarities, and differences in genetic and cellular changes associated with CDK1 in various tumors and tumor microenvironments. Our findings elucidate that *CDK1* expression increases in more than 20 human tumors and is highly correlated with oncogenic signature gene sets, biological pathways, immune cell infiltration, tumor mutational burden, microsatellite instability, and lower survival rate across multiple tumors. Targeting CDK1 may provide a novel and effective strategy for cancer immunotherapy.

**Abstract:**

Cyclin-dependent kinase 1 (CDK1) is essential for cell division by regulating the G2/M phase and mitosis. CDK1 overexpression can also promote the development and progression of a variety of cancers. However, the significance of CDK1 in the formation, progression, and prognosis of human pan-cancer remains unclear. In the present study, we used The Cancer Genome Atlas database, Clinical Proteomic Tumor Analysis Consortium, Human Protein Atlas, Genotype-Tissue Expression, and other well-established databases to comprehensively examine CDK1 genetic alterations and gene/protein expression in various cancers and their relationships with the prognosis, immune reactivities, and clinical outcomes for 33 tumor types. Gene set enrichment analysis was also conducted to examine the potential mechanisms of CDK1 in tumorigenesis. The data showed that *CDK1* mutation was frequently present in multiple tumors. CDK1 expression was significantly increased in various types of tumors as compared with normal tissues and was associated with poor overall and disease-free survival. In addition, CDK1 expression was significantly correlated with oncogenic genes, proteins, cellular components, myeloid-derived suppressor cell infiltration, ESTMATEScore, and signaling pathways associated with tumor development and progression and tumor microenvironments. These data indicate that CDK1 could serve as a promising biomarker for predicting tumor prognosis and a potential target for cancer treatment.

## 1. Introduction

Cyclin-dependent kinases (CDKs) are families of protein kinases that are critically involved in cell division, migration, gene transcription, and other important cellular and molecular processes [1]. The best-known CDK for cell cycle regulation is CDK1, which was first described in the genetic screening of the cell division cycle in yeast by Hartwell in 1970 [2,3]. CDK1 is also known as cell division cycle 2 homolog A, p34 protein kinase, p34, CDC2, and CDC28A [4]. It is a 34 kDa intracellular protein and expressed in many normal cells and organ systems, especially bone marrow, lymphoid tissues, gastrointestinal tract, skin, kidney, and testis [5]. CDK1 is an important catalytic subunit of the highly conserved protein kinase complex known as M-phase promoting factor (MPF) in both meiosis and mitosis [6]. The primary function of CDK1 is to critically regulate the eukaryotic cell cycle by modulating the centrosome cycle as well as mitotic onset, promoting G2-M transition, and regulating the G1 progress and G1-S transition via association with multiple interphase cyclins [7]. Increased CDK1 expression has been observed in tumor cells, including pancreatic ductal adenocarcinoma, esophageal squamous cell carcinoma, and hepatocellular carcinoma [8,9,10]. Inhibiting CDK1 or CDK1-related signaling pathways could increase the efficacy of cancer treatment by overcoming immune or apoptotic resistance [10,11,12]. In addition, CDK1-specific deletion in the liver decreases tumorigenesis [13]. Although extensive evidence reveals a close relationship between CDK1 and cancer, there is no comprehensive pan-cancer analysis of CDK1 yet.

The development, progression, and prognosis of tumors are very complicated and involve significant changes in the expression and/or mutations of many diverse genes. A large number of human tumors have been screened and evaluated by The Cancer Genome Atlas (TCGA), including ACC, BLCA, BRCA, CESC, CHOL, COAD, DLBC, ESCA, GBM, HNSC, LGG, LIHC, LUAD, LUSC, OV, PAAD, READ, SARC, STAD, THYM, UCEC, UCS, SKCM, KICH, KIRC, KIRP, LAML, MESO, PCPG, PRAD, TGCT, THCA, and UVM (the full names are listed in Table S1) [14,15]. Pan-cancer analysis based on the TCGA database has provided information on the cellular and molecular abnormalities of DNA, RNA, and proteins, their roles, and their relations to clinical prognosis for the 33 tumor types listed above. There are complex interactions among multiple genes and proteins for the initiation and progression of tumors. Gene set enrichment analysis and protein–protein interaction (PPI) networks can provide information on CDK1-related genes and proteins that are associated with oncogenic signaling pathways.

The tumor microenvironment (TME), including immune cells, stromal cells, extracellular matrix, and blood vessels, is critical for the fate of tumors [16]. It has been documented that CDK1 links communications between the TME and tumor cells in hepatocellular carcinoma [17]. JUN-dependent immune checkpoint expression was inhibited by blocking CDK1/2/5 in pancreatic cancer in response to interferon gamma [11]. Targeting immune checkpoints by blocking co-inhibitory molecules or activating co-stimulatory molecules has emerged as one of the important options in cancer treatment [18]. Microsatellite instability (MSI) and tumor mutational burden (TMB) function as predictive biomarkers for favorable outcomes of cancer immunotherapy [19]. However, the role of CDK1 in TME, immune checkpoints, MSI, and TMB is unclear.

The present study was designed to use a pan-cancer analysis to understand the role of CDK1 in human tumor development, progression, and clinical outcomes, as well as potential signaling pathways. The objectives were: (1) to investigate CDK1 gene alterations and gene and protein expression in various tumors; (2) to determine the correlation between CDK1 expression and clinical outcome; (3) to study CDK1-related genes and proteins and their roles in tumorigenesis using gene enrichment analysis; and (4) to determine the role of CDK1 in immune reactivity and immunotherapy.

## 2. Materials and Methods

### 2.1. Analyses of Genetic Alterations

The cBioPortal database (https://www.cbioportal.org/, accessed on 12 March 2022) was used to analyze genetic alterations in the *CDK1* gene [20]. The “Cancer Types Summary” in the “TCGA PanCancer Atlas Studies” module of cBioPortal was selected to obtain cancer genomic data of *CDK1*, including copy number alterations (CNAs), alteration frequency, and tumor entity summary. Then, the “mutation” module was selected, and the alteration/mutation types and case numbers were identified and analyzed in a diagram of *CDK1* alteration sites.

### 2.2. Analyses of Gene Expression

The difference in the expression of *CDK1* between tumor and normal tissues was examined from the “Box Plot” module in the “Expression analysis” of Gene Expression Profiling Interactive Analysis version 2 (GEPIA2) database (http://gepia2.cancer-pku.cn/, accessed on 12 March 2022) with the default parameters, log2FC cutoff = 1, *p*-value cutoff = 0.01, and matched TCGA normal and GTEx data [21]. The “Pathological Stage Plot” module of “Expression DIY” in the GEPIA2 website was used to generate violin plots of *CDK1* gene expression in tumors at different disease stages. The profiles of *CDK1* gene expression in different tissues were acquired from the Human Protein Atlas (HPA) database (https://www.proteinatlas.org/, accessed on 13 March 2022).

### 2.3. Analyses of Protein Expression

The Clinical Proteomic Tumor Analysis Consortium (CPTAC) Confirmatory/Discovery module of the UALCAN database (http://ualcan.path.uab.edu/analysis-prot.html, accessed on 12 March 2022) was used to acquire total CDK1 protein expression in human tumors and normal tissues [22]. The HPA database was used to obtain the profiles of CDK1 protein expression in different tissues and pathological sections for analysis.

### 2.4. Analyses of Prognosis and Survival

The “Survival Map” and “Survival analysis” modules of GEPIA2 were selected to evaluate the relationship between overall survival (OS) and disease-free survival (DFS) and *CDK1* expression in tumors based on the TCGA database. According to the median value of *CDK1* expression with 50% cutoff-high and 50% cutoff-low, patients were divided into high and low *CDK1* expression groups. The R packages “rms” and “survival” in the R software (version 3.6.3, R Foundation for Statistical Computing, Vienna, Austria) were used for prognosis and survival analysis using prognostic nomograms [23].

### 2.5. Analyses of CDK1-Related Genes and Proteins

The “Similar Genes Detection” module of GEPIA2 was used to examine the top 100 *CDK1*-correlated target genes based on the TCGA tumor cohort. Then, the top 5 genes were selected to conduct Pearson correlation analysis with *CDK1* using the “correlation analysis” module of GEPIA2. Additionally, a heatmap of the correlation between the selected top 5 genes and *CDK1* in different tumors was analyzed using Spearman’s correlation test in the “Gene_Corr” module of TIMER2.

Protein–protein interactions with CDK1 were analyzed using the STRING tool (https://string-db.org/, accessed on 13 March 2022). An estimated 50 experimentally determined proteins that were related to CDK1 were identified in the PPI network. The corresponding genes of these 50 proteins were named *CDK1*-interacted genes for analysis in the present study.

Intersection analysis of *CDK1*-interacted and *CDK1*-correlated genes was performed using Venn Diagram (http://bioinformatics.psb.ugent.be/webtools/Venn/, accessed on 13 March 2022) [24]. Gene Ontology (GO) enrichment analysis (biological processes, cellular components, and molecular function) and Kyoto Encyclopedia of Genes and Genomes (KEGG) pathway analysis were conducted using the R package “clusterProfiler” in the R software after combining with the datasets of *CDK1*-related genes.

### 2.6. Analyses of Gene Set Enrichment

Gene set enrichment analysis (GSEA) based on the TCGA tumor database between *CDK1* high- and low-expression groups was performed to determine the biological and oncogenic signaling pathways. MSigDB H (hallmark gene sets) and C6 (oncogenic signature gene sets) enrichment analyses were conducted using the R package “clusterProfiler” in the R software [25]. Enrichment was considered significant when |NES| > 1, *p*.adjust < 0.05, and FDR < 0.25.

### 2.7. Analyses of Cancer Immune Reactivity

The ESTIMATE, Immune, and Stromal scores of *CDK1* in tumors were conducted using SangerBox (http://sangerbox.com/, accessed on 13 March 2022) [26]. The “Immune Association” module of TIMER2 was selected to explore the association between *CDK1* expression and immune infiltration of myeloid-derived suppressor cells (MDSCs) in different tumors in the TCGA database. In addition, correlations between various immune checkpoints, MSI, and TMB with *CDK1* expression in various tumors in the TCGA database were analyzed using SangerBox. The R package “GSVA” in the R software was used to determine the correlation between *CDK1* expression and infiltration of 24 common immune cells in tumors of the TCGA dataset by single-sample GESA (ssGESA) [27,28]. Cellular heterogeneity of *CDK1* expression in tumors was conducted using Cancer Single-cell Expression Map (https://ngdc.cncb.ac.cn/cancerscem/index, last accessed on 30 April 2022) [29].

### 2.8. Statistical Analysis

The Wilcoxon test was used to analyze data on gene expression from GEPIA2 based on TCGA and GTEx databases. Student’s *t*-test was used to analyze protein expression data from UALCAN. The log-rank test was used to analyze the survival data from GEPIA2. The R package “clusterProfiler” in the R software was used to analyze GO, KEGG pathway, MSigDB H, and C6 enrichment. Purity-adjusted Spearman’s rho was used to analyze correlation data from TIMER2. Pearson’s correlation coefficients were used to analyze correlation data from SangerBox. A difference was reported as statistically significant when *p* < 0.05.

## 3. Results

### 3.1. Genetic Variation Analysis of CDK1 in Tumors

**Figure 1:**
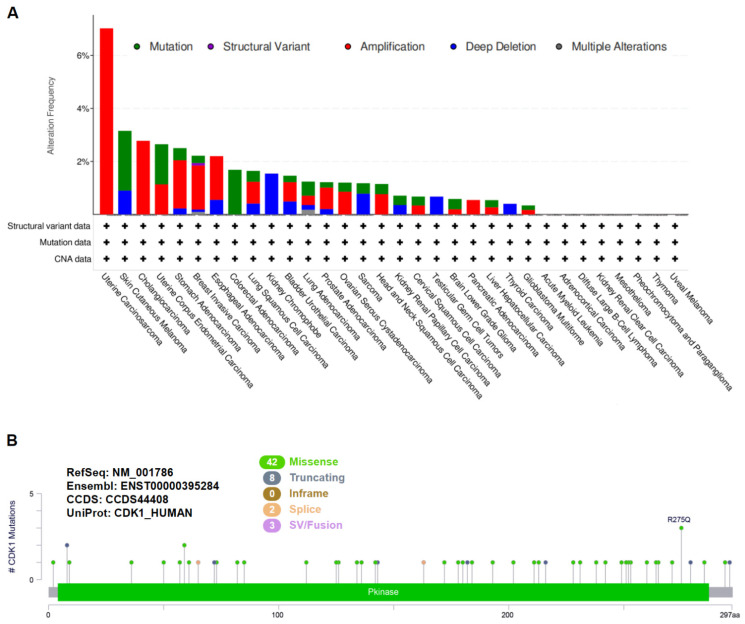
CDK1 genetic alterations in different tumors of TCGA database. (**A**) The alteration frequencies and types in various tumors. (**B**) CDK1 mutation types, sites, and case numbers.

*CDK1* genetic alterations in various tumors in the TCGA database were identified and are summarized in Figure 1A. The highest frequency of *CDK1* genetic alteration (7.02%) was observed in patients with uterine carcinosarcoma, with amplification as the primary genetic alteration. Mutation and deep deletion were observed in cutaneous melanoma, which had the second highest frequency of *CDK1* genetic alterations (3.15%). The mutation types, sites, and case numbers of *CDK1* in different tumors are shown in Figure 1B. The most common type of mutation was missense, followed by truncation. The transcriptional expression of *CDK1* in structural variants of amplification, gain, diploid, shallow deletion, and deep deletion in various tumors, such as BRCA, STAD, SKCM, COAD, UCEC, and USC, is shown in Figure S1.

### 3.2. CDK1 Gene and Protein Expression in Tumors

**Figure 2:**
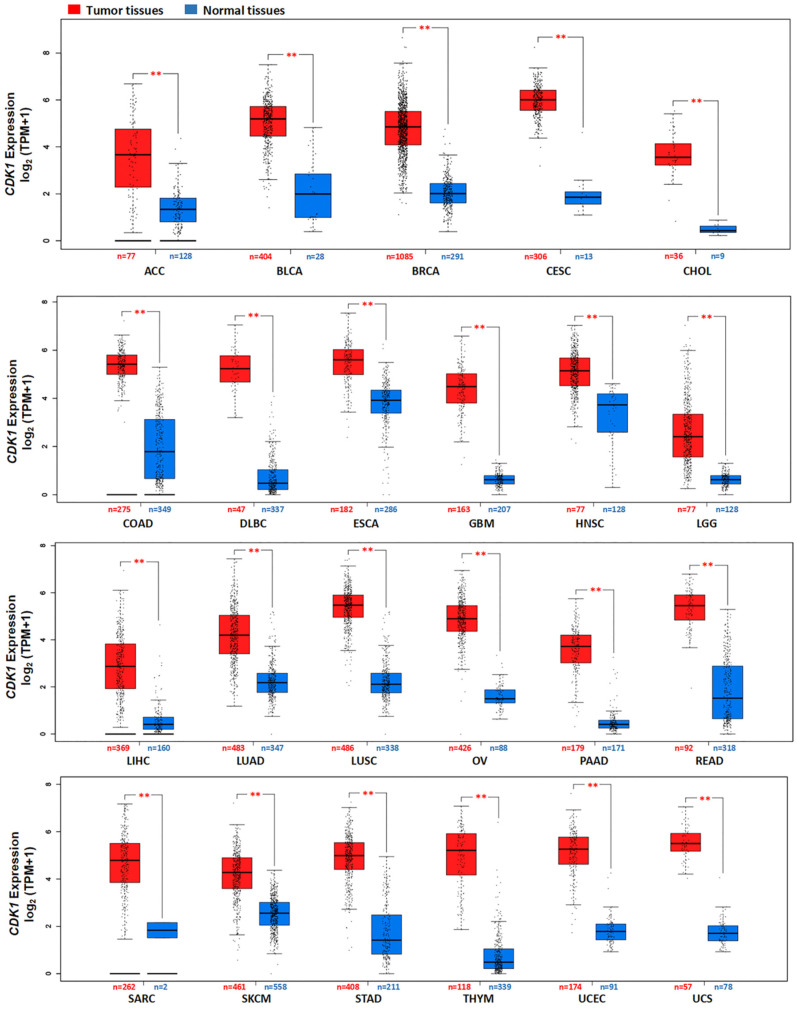
*CDK1* mRNA expression in various tumors and normal tissues. The expression of *CDK1* in some tumors from the TCGA database was significantly increased as compared to normal tissues from the GTEx database; Expression data were log_2_ (TPM + 1) transformed for plotting. ** *p* < 0.01, Wilcoxon test. TPM: transcripts per million.

The gene expression of *CDK1* in various normal tissues is shown in Figure S2A. It was found that the highest *CDK1* RNA expression was observed in the bone marrow and lymphoid tissue. The consensus dataset consisting of TCGA and GTEx databases revealed that the level of *CDK1* gene expression was dramatically increased in most tumor tissues when compared to normal tissues, including ACC, BLCA, BRCA, CESC, CHOL, COAD, DLBC, ESCA, GBM, HNSC, LGG, LIHC, LUAD, LUSC, OV, PAAD, READ, SARC, SKCM, STAD, THYM, UCEC, and UCS (Figure 2 and Figure S3). There was a significant correlation between the gene expression of *CDK1* and the stages of some tumors, such as KIRC, BRCA, KIRP, LUAD, ACC, and KICH (Figure S4). However, the level of *CDK1* gene expression was significantly decreased in LAML when compared to normal tissues (Figure S5).

**Figure 3:**
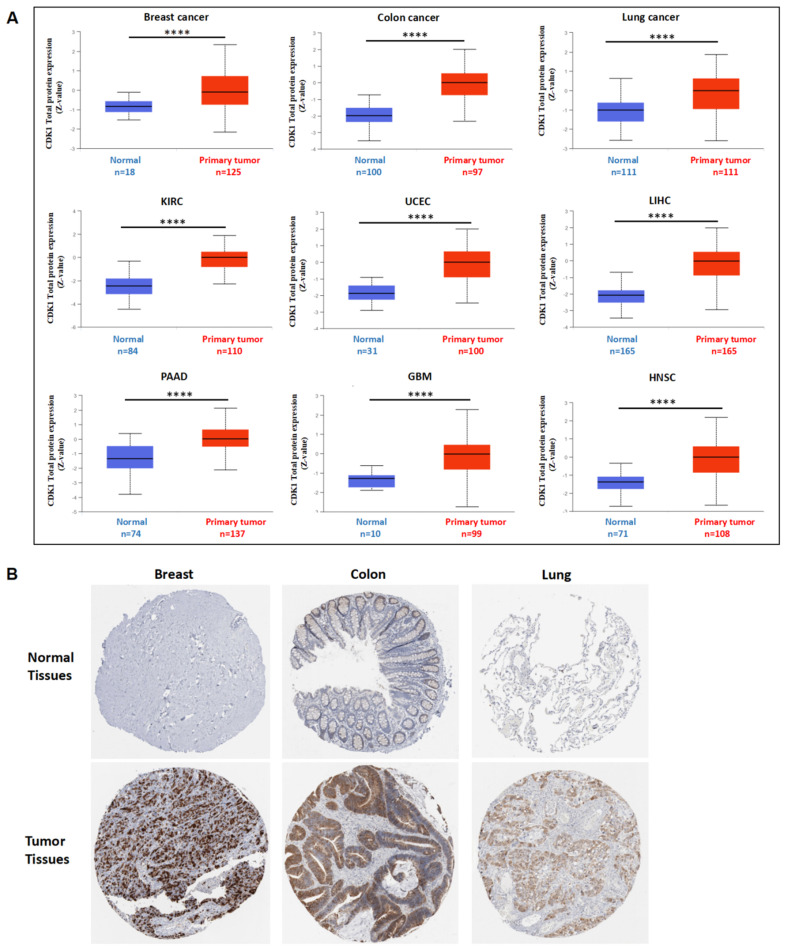
CDK1 protein expression in various tumors and normal tissues. (**A**) The total CDK1 protein expression was significantly higher in primary tumors as compared to normal tissues from the CPTAC database; for a given cancer type, Z-value represents the standard deviation from the median across samples. **** *p* < 0.0001, Student’s *t*-test. (**B**) Immunohistochemistry staining images of CDK1 in human breast, colon, and lung from the HPA database.

The highest level of CDK1 protein expression was observed in the testis, lymph nodes, and tonsil (Figure S2B). The total protein expression of CDK1 was significantly higher in some primary tumors, including breast cancer, colon cancer, lung cancer, KIRC, UCEC, LIHC, PAAD, GBM, and HNSC, as compared to normal tissues (Figure 3A). Immunohistochemistry staining also demonstrated a significant increase in CDK1 protein in breast cancer, colon cancer, and lung cancer tissues from patients but very minimal CDK1 protein in healthy human tissues (Figure 3B).

### 3.3. Survival Analysis

**Figure 4:**
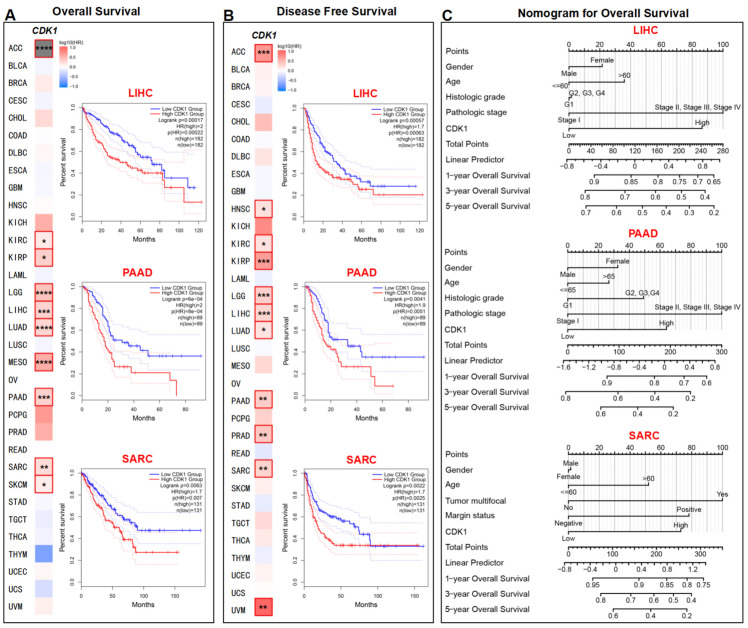
Correlation between *CDK1* expression and survival in different tumors. Survival maps of different tumors and Kaplan–Meier curves of LIHC, PAAD, and SARC are given. High *CDK1* expression was correlated with (**A**) overall survival and (**B**) disease-free survival in multiple tumors from the TCGA database. (**C**) Nomograms predicting one-, three-, and five-year overall survival of LIHC, PAAD, and SARC. Variables include gender, age, histological grade, pathologic stage, tumor multifocal, margin status, and *CDK1* expression. * *p* < 0.05, ** *p* < 0.01, *** *p* < 0.001, **** *p* < 0.0001, log-rank test. HR: hazard ratio.

To investigate the relationship between *CDK1* expression and the prognosis of the tumor, the TCGA dataset was separated into two groups: one with high *CDK1* expression and the other with low *CDK1* expression. As shown in Figure 4A and Figure S6, tumors with high *CDK1* expression, including ACC, KIRC, KIRP, LGG, LIHC, LUAD, MESO, PAAD, SARC, and SKCM, were related to poor overall survival (OS) (*p* < 0.05). High *CDK1* expression was also associated with poor disease-free survival (DFS) for ACC, HNSC, KIRC, KIRP, LGG, LIHC, LUAD, PAAD, PRAD, SARC, and UVM (Figure 4B, *p* < 0.05). Nomograms were used to predict one-, three-, and five-year OS of LIHC, PAAD, and SARC. The corresponding clinical and pathological points for each patient, such as gender, age, histological grade, tumor stage, and expression of *CDK1*, were calculated and added together to obtain the total points. A higher number of total points served as a predictor for poorer OS. As shown in Figure 4C, high expression of *CDK1* contributed to a large portion of the total points for LIHC, PAAD, and SARC.

### 3.4. CDK1-Related Genes and Protein–Protein Interactions

**Figure 5:**
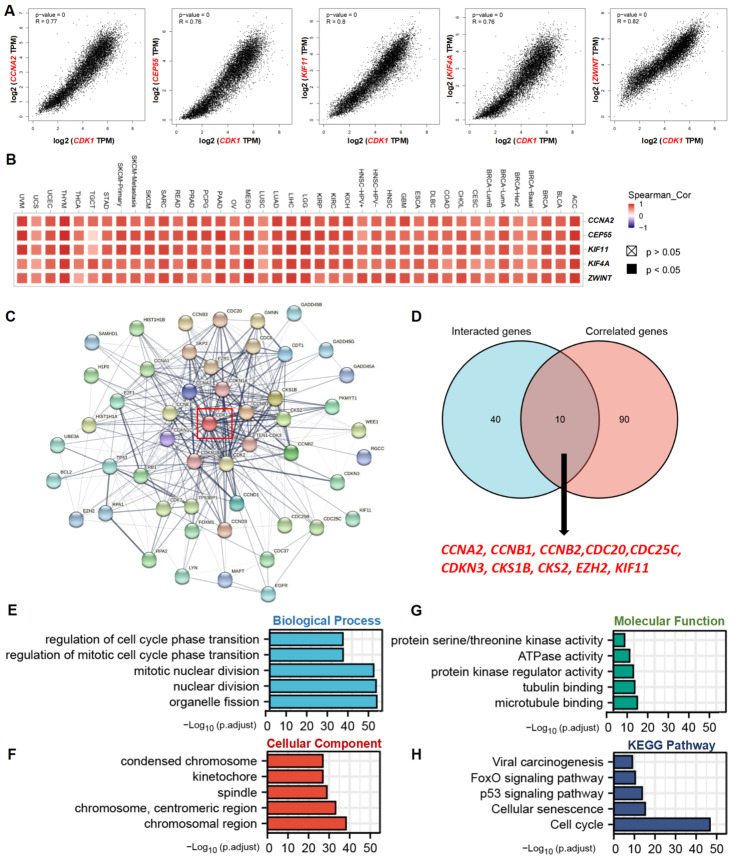
*CDK1*-related gene network, GO enrichment analysis, KEGG pathway analysis, and protein–protein interactions. (**A**) Top 5 *CDK1*-correlated genes in TCGA database, *CCNA2*, *CEP55*, *KIF11*, *KIF4A*, and *ZWINT*, Pearson’s correlation coefficients. (**B**) The heatmap reveals that *CDK1* expression was positively correlated with these five genes in all tumor types according to purity-adjusted partial Spearman’s rho values. (**C**) PPI network of 50 experimentally verified CDK1-interacted proteins. (**D**) An intersection analysis of the *CDK1*-interacted and *CDK1*-correlated genes. (**E**–**G**) The correlations between *CDK1* and biological processes, cellular components, and molecular functions according to GO enrichment analysis. (**H**) KEGG pathway analysis was performed based on the *CDK1*-interacted and *CDK1*-correlated genes.

*CDK1*-related gene analysis and protein–protein interaction (PPI) analysis were performed to explore the potential mechanisms of CDK1 in tumorigenesis. The top 100 *CDK1*-correlated genes were identified (Table S2), and the top five correlated genes were obtained and are shown in Figure 5A: cyclin A2 (*CCNA2*, R = 0.77), centrosomal protein 55 (*CEP55*, R = 0.76), kinesin family member 11 (*KIF11*, R = 0.8), kinesin family member 4A (*KIF4A*, R = 0.76), and ZW10 interacting kinetochore protein (*ZWINT*, R = 0.82). In addition, there was a significant positive correlation between these top five genes and *CDK1* in all tumor types from the TCGA database (Figure 5B). The top 50 *CDK1*-interacted genes were also identified (Table S2). Analysis of protein–protein interactions revealed a total of 50 proteins that experimentally interacted with CDK1, with the top five node degree proteins being: CDK2, TEN1-CDK3, CCNB1, CCNA2, and CDKN1 (Figure 5C).

Cross-analysis of *CDK1*-interacted genes and *CDK1*-correlated genes revealed 10 common member genes: *CCNA2*, *CCNB1*, *CCNB2*, cell division cycle 20 (*CDC20*), *CDC25C*, cyclin-dependent kinase inhibitor 3 (*CDKN3*), CDC28 protein kinase regulatory subunit 1B (*CKS1B*), enhancer of zeste 2 polycomb repressive complex 2 subunit (*EZH2*), and *KIF11* (Figure 5D). The GO enrichment analysis of the two combined datasets indicated that the *CDK1*-interacted or -correlated genes were related to the biological processes of organelle fission, nuclear division, mitotic nuclear division, and cell cycle regulation (Figure 5E); to the cellular structures of the spindle, chromosome, centromeric region, condensed chromosome, and kinetochore (Figure 5F); and to the cellular functions of tubulin/microtubule binding, ATPase activity, serine/threonine kinase activity, and protein kinase regulator activity (Figure 5G). In addition, KEGG pathway analysis showed that *CDK1* was involved in the regulation of cellular senescence, the cell cycle, viral carcinogenesis, p53 signaling, and FoxO signaling, thus potentially contributing to oncogenesis (Figure 5H).

### 3.5. Gene Set Enrichment Analysis (GSEA)

**Figure 6:**
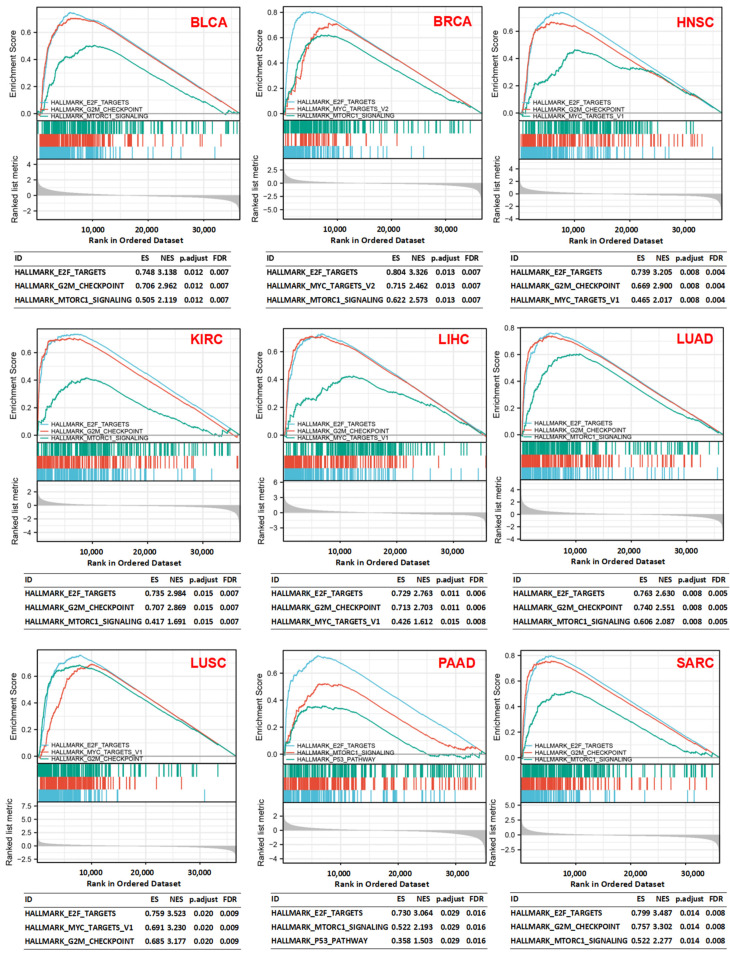
Hallmark gene set enrichment analysis (GSEA). “HALLMARK E2F TARGETS”, “HALLMARK G2M CHECKPOINT”, “HALLMARK MTORC1 SIGNALING”, “HALLMARK MYC TARGETS”, and the “HALLMARK P53 PATHWAY” enriched in the high *CDK1* expression group compared to the low *CDK1* expression group in various tumors. Each line represents one particular gene set with a unique color, with upregulated genes located on the left and the ones with the highest expression on the far left, while downregulated genes are shown on the right. NES: normalized enrichment score; FDR: false discovery rate.

**Figure 7:**
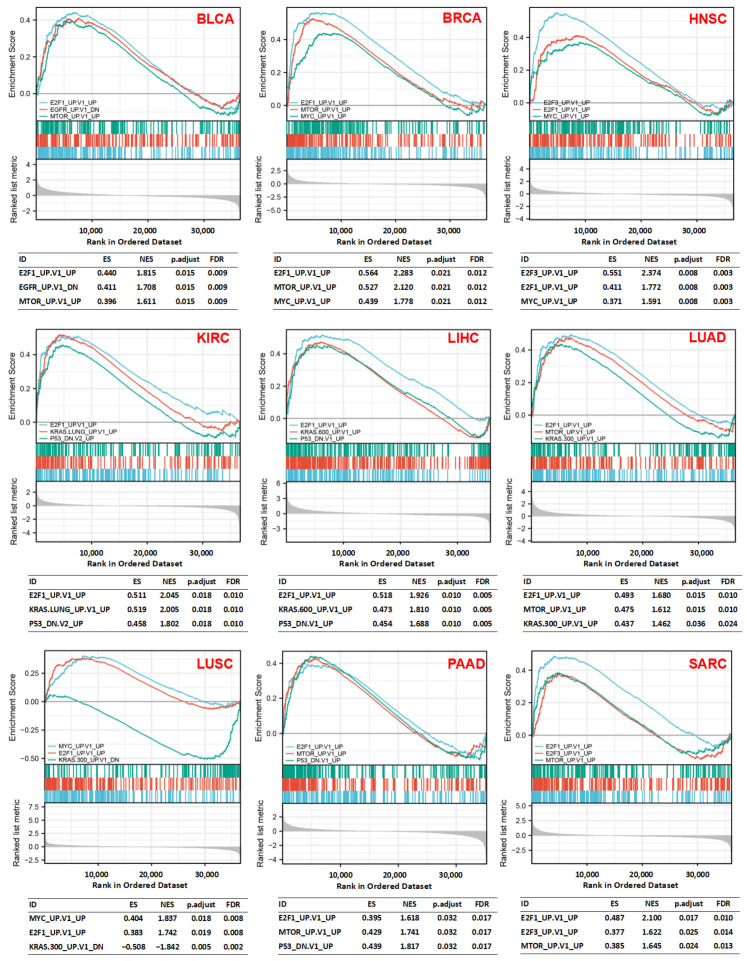
Oncogenic signature gene set enrichment analysis. *CDK1* expression was associated with various signature oncogenes. Each line represents one particular gene set with a unique color, and upregulated genes are located on the left, while down-regulated genes are displayed on the right, with the lowest ones on the right end. ES: enrichment score; NES: normalized enrichment score; FDR: false discovery rate.

Analysis of H hallmark gene sets and C6 oncogenic signature gene sets in the molecular signatures database (MSigDB) demonstrated that coherent expression and cellular pathways were often dysregulated in cancer cells based on the levels of *CDK1* expression. It was found that high *CDK1* expression was associated with cell-cycle-related targets of E2F transcription factors, the G2M DNA damage checkpoint, mTORC1 signaling, MYC target genes, and the P53 pathway in various tumors, such as BLCA, BRCA, HNSC, KIRC, LIHC, LUAD, LUSC, PAAD, and SARC (Figure 6). The C6 enrichment analysis showed that high expression of *CDK1* was associated with genes that were upregulated with the overexpression of oncogenes, such as *E2F1*, *MTOR*, *MYC*, *E2F3*, and *KRAS*, and genes that were upregulated with the knockdown of the tumor suppressor gene *P53*. However, low expression of *CDK1* was related to genes that were downregulated by the overexpression of an oncogenic form of *KRAS* (Figure 7).

### 3.6. CDK1 and Immune Reactivity

**Figure 8:**
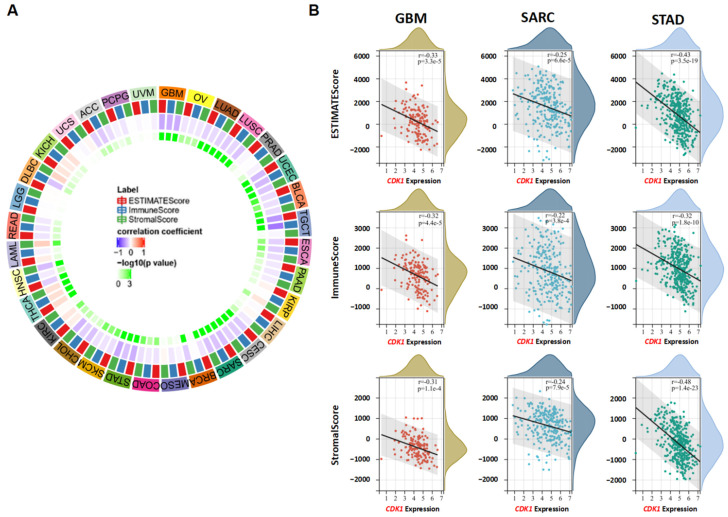
The association of *CDK1* expression with ESTIMATEScore, ImmuneScore, and StromalScore in tumors. (**A**) The heatmap shows that *CDK1* expression was significantly associated with these scores in some tumors. (**B**) Representative scatter plots of GBM, SARC, and STAD are shown. Pearson’s correlation coefficients.

The correlations between *CDK1* expression and the ESTIMATEScore, the ImmuneScore, and the StromalScore were estimated using SangerBox in diverse tumor types from the TCGA database (Table S3). The EstimateScore is the sum of the ImmuneScore and StromalScore. A lower ImmuneScore or StromalScore indicates a lower percentage of immune cells or stromal cells in the core and the invasive margin of the tumor [26]. It was found that *CDK1* expression was negatively correlated with the ESTIMATEScore, the ImmuneScore, and the StromalScore in GBM, UCEC, STAD, and SKCM (r < −0.3, *p* < 0.05) (Figure 8A). Conversely, *CDK1* expression was positively associated with these scores in KIPAN (KICH + KIRC + KIRP) (r > 0.3, *p* < 0.05). Representative scatter plots display a significant and negative correlation between the expression of *CDK1* and the ESTIMATEScore, the ImmuneScore, and the StromalScore in GBM, SARC, and STAD (Figure 8B).

**Figure 9:**
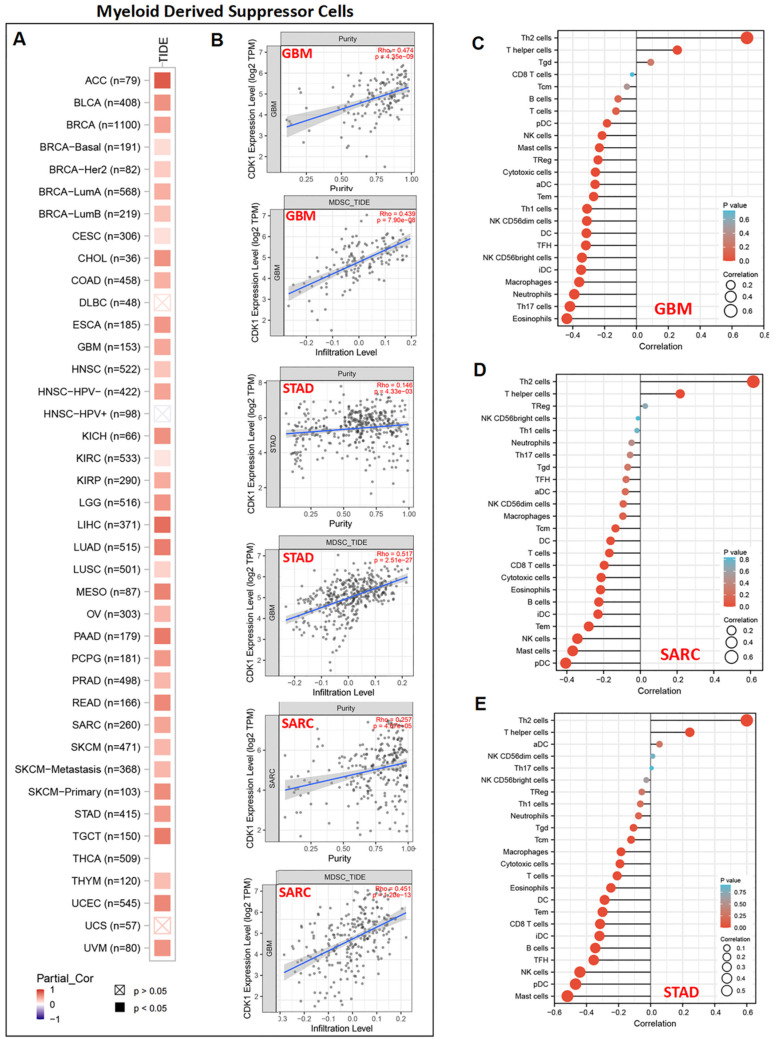
Association between *CDK1* expression and immune infiltration. (**A**,**B**) The expression of *CDK1* was positively related to MDSC infiltration in almost all of the tumors. Purity-adjusted Spearman’s rho values, with TIDE algorithm. (**C**–**E**) The representative lollipop chart shows that *CDK1* expression was significantly related to the infiltration of various innate and adaptive immune cells in GBM, SARC, and STAD.

**Figure 10:**
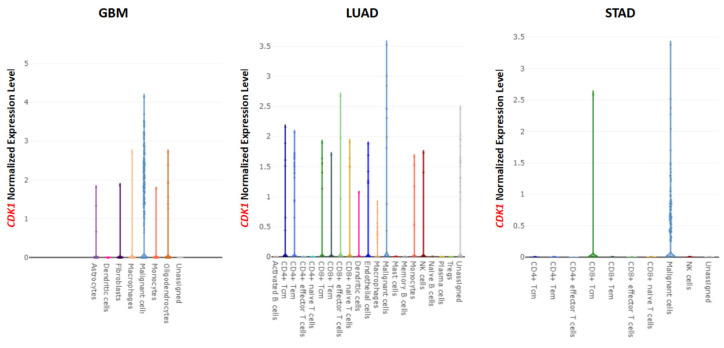
Single-cell data analysis of *CDK1* expression in tumors. *CDK1* was mainly expressed in malignant cells and also abundantly expressed in some immune cells in GBM, LUAD, and STAD.

The correlation between MDSC infiltration and the expression of *CDK1* was evaluated based on the TCGA database. As shown in Figure 9A, a significant and positive correlation between *CDK1* expression and MDSC infiltration was observed in almost all tumors, except for DLBC, HNSC-HPV+, and UCS. Representative scatter plots show that the purity and infiltration levels of MDSCs were significantly increased when the expression of *CDK1* was increased in GBM, STAD, and SARC (Figure 9B). In addition, the representative lollipop chart of immune infiltration demonstrates that the expression of *CDK1* in GBM, SARC, and STAD was positively related to Th2 cells and negatively associated with most of the immune cells, such as eosinophils, pDC, and mast cells (Figure 9C–E). Single-cell data showed that *CDK1* was mainly expressed in malignant cells (Figure 10).

**Figure 11:**
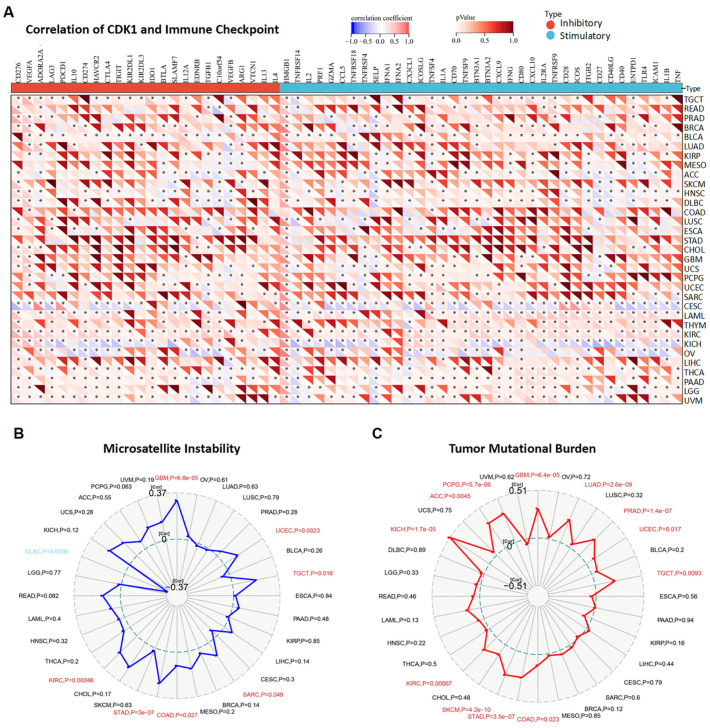
Correlations of immune checkpoints, MSI, and TMB with *CDK1* expression. (**A**) *CDK1* expression was significantly associated with many immune checkpoint genes in multiple tumors. (**B**,**C**) The radar chart shows that the expression of *CDK1* was significantly and positively related to MSI and TMB in several tumors. * *p* < 0.05, Pearson’s correlation coefficients.

Immune checkpoint genes (ICGs) are among the critical immunotherapy targets for cancer treatment. *CDK1* expression was positively related to many inhibitory ICGs, such as cluster of differentiation 276 (*CD276*), vascular endothelial growth factor A (*VEGFA*), cytotoxic T-lymphocyte-associated protein 4 (*CTLA4*), and programmed cell death protein 1 (*PDCD1*) in various tumors (Figure 11A). Notably, high mobility group box 1 (*HMGB1*) was significantly and positively related to *CDK1* in 31 different types of tumors. A series of stimulatory ICGs, such as *CD40*, TNF receptor superfamily member 14 (*TNFRSF14*), and selectin P (*SELP*), were negatively linked with *CDK1* in some tumors (OV, KICH, and CESC) (Figure 11A). Analysis of the correlations between the expression of *CDK1* and MSI/TMB in tumors in the TCGA database showed that *CDK1* expression was positively related to MSI in GBM, UCEC, TGCT, SARC, COAD, STAD, and KIRC, whereas *CDK1* expression was negatively linked with MSI in DLBC (Figure 11B). In addition, high expression of *CDK1* was associated with high TMB in GBM, LUAD, PRAD, UCEC, TGCT, COAD, STAD, SKCM, KIRC, KICH, ACC, and PCPG (Figure 11C).

## 4. Discussion

The present pan-cancer analysis demonstrated: (1) *CDK1* genetic alterations, including amplification, mutation, and deep deletion, were observed in a variety of cancers; (2) the gene expression of *CDK1* was significantly increased in many tumor tissues based on the TCGA database, including ACC, BLCA, BRCA, CESC, CHOL, COAD, DLBC, ESCA, GBM, HNSC, LGG, LIHC, LUAD, LUSC, OV, PAAD, READ, SARC, SKCM, STAD, THYM, UCEC, and UCS, as compared to normal tissues; (3) high total protein expression of CDK1 was observed in a variety of primary tumors from the CPTAC database, including breast cancer, colon cancer, lung cancer, KIRC, UCEC, LIHC, PAAD, GBM, and HNSC; (4) poor OS and DFS were associated with high *CDK1* expression in multiple tumors, such as ACC, KIRC, KIRP, LGG, LIHC, LUAD, PAAD, and SARC; (5) the top five *CDK1*-correlated genes were *CCNA2*, *CEP55*, *KIF11*, *KIF4A*, and *ZWINT*: these genes were positively and significantly correlated with *CDK1* in all tumor types; (6) *CDK1*-interacted or -correlated genes were closely associated with “cell cycle”, “organelle fission”, “nuclear division”, and other cellular functions related to carcinogenesis; (7) high *CDK1* expression was associated with genes in E2F, G2M, mTORC1, MYC, and P53 pathways in various tumors, such as BLCA, BRCA, HNSC, KIRC, LIHC, LUAD, LUSC, PAAD, and SARC; (8) *CDK1* expression was significantly correlated with the ESTIMATEScore, the ImmuneScore, and the StromalScore in some tumors; (9) there was positive correlation between *CDK1* expression and MDSC infiltration in almost all of the tumor types from the TCGA database; (10) *CDK1* was positively related to Th2 cells in GBM, SARC, and STAD and negatively associated with eosinophils, pDC, mast cells, and other immune cells in tumors; (11) *CDK1* was correlated with several immune checkpoints in various tumors, including *CD276*, *VEGFA*, *CTLA4*, *PDCD1*, *CD40*, *TNFRSF14*, and *SELP*; and (12) MSI and TMB were also significantly correlated with the expression of *CDK1*. Taken together, these data suggest that CDK1 is highly related to tumorigenesis and could be a potential target for tumor diagnosis and treatment.

CDK1, one of the serine/threonine kinases, plays a critical role in regulating the centrosome cycle and mitotic onset. During G2 and early mitosis, CDK1–cyclin complexes are activated and phosphorylate more than 70 substrates to promote the separation of centrosomes, collapse of the nuclear envelope, and condensation of chromosomes [30]. Notably, CDK1 can balance cell proliferation and protein synthesis [31]. Mutations in various genes and uncontrolled cell division can lead to the development of tumors. Indeed, the data from the present study showed that amplification, mutation, deep deletion, and other genetic alterations of *CDK1* were present in multiple tumors from the TCGA cohort, such as UCS, SKCM, CHOL, STAD, and BRCA. Previous studies have demonstrated that the expression of CDK1 is increased in some human tumors, and an increase in CDK1 expression is associated with a poor prognosis for hepatocellular carcinoma and pancreatic ductal adenocarcinoma [10,32,33]. Tumorigenic potential and tumor-initiating capacity are significantly increased in melanoma cells with CDK1 overexpression due to its interaction with the pluripotent stem cell transcription factor Sox2 [34]. Clinically, CDK1-mediated phosphorylation of human telomerase reverse transcriptase (hTERT) at T249 is closely related to aggressive and advanced cancers [35]. The phosphorylation of transcription factor CP2-like 1 (TFCP2L1) by CDK1 at Thr177 promotes bladder carcinogenesis, and the tumorigenic potency of bladder cancer cells was reduced in a xenograft model when the level of TFCP2L1 phosphorylation was decreased [36]. In the present study, based on the TCGA database, both gene and protein expression of CDK1 were significantly higher in some tumors than in normal tissues, and CDK1 activity was associated with a poor prognosis in patients with some tumors, such as KIRC, UCEC, LIHC, PAAD, GBM, LUAD, and HNSC.

Multiple changes in genes and proteins are frequently observed in tumors, and the interactions among these genes and proteins are important for tumor development and progression. Thus, the role of CDK1 in carcinogenesis would be associated with many other genes and proteins. Indeed, the present study showed that *CCNA2*, *CEP55*, *KIF11*, *KIF4A*, and *ZWINT* were the top five genes that were significantly and positively correlated with *CDK1* in all tumor types from the TCGA database. CCNA2 activates CDK1 during the S phase and early mitosis and promotes nuclear accumulation of cyclin B1-CDK1 [37,38,39]. Hyperactivation of CCNA2-CDK1 could cause abnormal replication in the early S phase, while CCNA2 deletion delays nuclear envelope breakdown and suppresses tumor formation [39,40,41]. Increased co-expression of CCNA2 and CDK1 has been observed in hepatoblastoma, and inhibiting the expression of CCNA2 and CDK1 attenuates the proliferative, migrative, and invasive capacities of both HepG2 and HuH-6 cells [42]. CEP55, a centrosomal protein, regulates cytokinesis and is upregulated in tumorigenesis [43]. A recent study has shown that CEP55 expression is increased in the tissue of colorectal cancer (CRC) and promotes the proliferation of CRC cells through interactions with p53/p21 signaling proteins [44]. CEP55 deletion can prime premature CDK1/cyclin B activation and mitotic cell death, thus sensitizing breast cancer cells to antimitotic drugs [43]. KIF11, a mitotic kinesin, is involved in the formation and maintenance of the bipolar spindle. Increased KIF11 expression has been reported in a variety of tumors, such as HCC, GBM, CRC, and gallbladder cancer (GBC), and affects the regulation of various signaling pathways, including ERBB2/PI3K/AKT and p53/GSK3β [45,46,47,48]. Blocking KIF11 with siRNA can inhibit the protein expression of Cyclin B1 and CDK1, thus arresting the cell cycle in the G2/M phase [45]. KIF4A is closely related to prostate cancer, liver cancer, and lung cancer through the regulation of spindle formation, centrosome assembly, chromosome concentration and separation, and DNA damage repair [49,50,51]. Chromosome binding and assembly during early mitosis are dependent on CDK1-mediated phosphorylation of KIF4A at S1186 [52]. ZWINT, a kinetochore-associated protein, is known to be critically involved in centromere function and cell growth and is required for the spindle assembly checkpoint [53]. CDK1 expression can be modified by ZWINT to promote HCC progression with increased tumor size and number [54]. Understanding the function of CDK1-related genes and their interactions may provide a novel and effective therapeutic strategy for cancers.

In the present study, GO analysis revealed that *CDK1* was related to genes involved in the chromosome, spindle, and kinetochore and genes that regulate cell cycle phase transition, microtubule binding, kinase activity, and cell division. The KEGG analysis also showed that *CDK1*-related genes were involved in the cell cycle, as well as P53 and FoxO signaling. According to GSEA, E2F transcription factors, the G2/M DNA damage checkpoint, MYC targets, mTORC1, and P53 signaling were associated with high *CDK1* expression. These results suggest that CDK1-mediated mechanisms in the cell cycle are important for cell proliferation and tumor initiation. It is known that E2F and MYC activities are highly regulated, and p53 activity is suppressed in tumors with enhanced cell growth and proliferation [55,56,57]. Cyclins and CDKs are upregulated during the cell cycle, and the progression of the cell cycle can be achieved by MYC-induced CDK1 activation [58]. Tumorigenesis induced by silencing p53 is attenuated after CDK1 deletion in the liver [13]. To avoid the accumulation and transmission of genetic errors during cell division, cell cycle checkpoints slow cell cycle progression and induce cell cycle exit or cell death [59]. Double-strand DNA breaks can activate DNA damage checkpoints, which depend on the checkpoint protein kinase ataxia telangiectasia mutated (ATM). In S and G2 phases, CHK2 and WEE1 can inhibit CDK1 activity to prevent mitotic entry [59]. However, CDK1 can phosphorylate the DNA damage signaling protein 53BP1 and drive cell cycle reentry by terminating the ATM-CHK2 branch of the G2/M checkpoint [60]. There are two functionally distinct mTOR complexes: one is rapamycin-sensitive (mTORC1), and the other is relatively rapamycin-resistant (mTORC2). Enhanced tumor formation, proliferation, and metastasis could be associated with mTORC1 overactivation [61]. Temsirolimus and everolimus are FDA-approved mTOR inhibitors for kidney or breast cancer. The inhibition of one signaling pathway may result in feedback activation of other signaling pathways. Blocking mTOR with rapamycin can cause CDK1 activation and further cell cycle progression [62]. Tumor development processes are complex, involving numerous signaling pathways. Therefore, more studies are welcomed to explore the mechanisms of CDK1 in tumorigenesis.

One of the major challenges in cancer therapy is drug resistance. Antimitotic drugs are usually used as the first-line treatment in patients with cancer. However, drug resistance decreases the efficacy of these drugs. Chromosomal instability (CIN) and aneuploidy are often observed in tumor cells that are resistant to antimitotic drugs. The CDK1-related gene CEP55 can protect aneuploid cells from death, while targeting CEP55 may sensitize cells to microtubule inhibitors [63]. It has been reported that cyclin B1/CDK1-mediated mitochondrial bioenergetics plays a role in tumor cell survival and the reduction in anticancer efficacy [64]. Inhibition of CDK1 has been shown to reverse Paclitaxel-induced resistance in ovarian cancer cells and 5-FU-induced resistance in colorectal cancer [65,66]. Immunotherapy can be combined with antimitotic drugs to treat cancer by boosting or modulating the immune system. MDSCs expand and negatively regulate the immune response in cancer, making the tumor resistant to immunotherapy and thus promoting tumor cell proliferation and invasion [67,68]. Increased levels of MDSCs were observed in patients with STAD or GBM and associated with advanced stages and poor prognoses of cancers [69,70]. Based on the TCGA database, the data from the present study indicated that *CDK1* expression was positively related to MDSC infiltration in almost all tumor types (except for DLBC, HNSC-HPV+, and UCS). The expression of *CDK1* was negatively correlated with the ImmuneScore, which reflects in situ T-cell infiltration in a variety of tumors. Indeed, most of the innate and adaptive immune cells are negatively related to *CDK1* expression in GBM, SARC, and STAD. Data on the role of Th2 cells in tumors have been inconsistent. Some studies show that Th2 contributes to antitumor immunity, while others show that Th2 promotes tumor growth and metastasis [71]. A positive correlation between *CDK1* and Th2 cells was observed in GBM, SARC, and STAD in the present study. However, the specific mechanisms of CDK1 and Th2 cells in tumorigenesis are not clear at this point.

Immune checkpoint inhibitors (ICIs) are one of the immunotherapies that work by promoting immune cell responses in cancer treatment. The present study showed that *CD276*, *CTLA4*, and *PDCD1* (*PD1*) were positively related to *CDK1* expression in some tumors in the TCGA database. Inhibitory checkpoints CTLA4 and PDCD1 are commonly found in activated T cells, and their inhibitors have been used in patients with tumors, including melanoma, bladder cancer, renal cell carcinoma, non-small cell lung cancer, Hodgkin lymphoma, MSI-high colorectal carcinoma, and Merkel cell carcinoma [72,73]. However, resistance to ICIs develops during treatment. The treatment efficacy of ICIs can be predicted by tumor MSI and TMB. The objective response rate to ICIs in 27 cancer types is positively correlated with TMB, and MSI-high is also used to define the antitumor efficacy of ICIs [74,75]. The present study also demonstrated that the expression of *CDK1* was significantly and positively correlated with MSI and TMB in multiple tumors in the TCGA database. These results suggest that CDK1 could be an effective target for immunotherapy and for the treatment of drug-resistant cancers. Alsterpaullone, Flavopiridol, and RO-3306 are CDK1 inhibitors and have been shown to increase drug efficacy and decrease tumor growth [10,65,76]. Future studies are required to clarify the role of CDK1 in cancer cell proliferation and invasion and to design specific CDK1 inhibitors to improve anticancer efficacy and reduce side effects.

## 5. Conclusions

The present pan-cancer analysis of CDK1 demonstrates that CDK1 is closely related to a variety of tumors, including ACC, BLCA, BRCA, CESC, CHOL, COAD, DLBC, ESCA, GBM, HNSC, LGG, LIHC, LUAD, LUSC, OV, PAAD, READ, SARC, SKCM, STAD, THYM, UCEC, and UCS. CDK1 is critically involved in the regulation of the “cell cycle”, “organelle fission”, and “nuclear division” and associated with genes in E2F, G2M, mTORC1, MYC, and P53 pathways. A poor clinical prognosis is observed in cancer patients with high *CDK1* expression. The negative correlations between *CDK1* and the ESTIMATEScore, the ImmuneScore, and the StromalScore, as well as the positive correlations between *CDK1* expression and MDSC infiltration, MSI, and TMB across multiple tumors, suggest that CDK1 may potentially serve as a novel and effective target for cancer immunotherapy.

## Data Availability

Not applicable.

## Supplementary Materials

The following supporting information can be downloaded at: https://www.mdpi.com/article/10.3390/cancers14112658/s1, Figure S1: The transcriptional expression of *CDK1* in different types of structural variants in various tumors. Figure S2: RNA and protein expression of CDK1 in different normal tissues based on the consensus datasets of the HPA and GTEx databases. Figure S3: The gene expression of *CDK1* in different cancers based on the TCGA database. Figure S4: The correlation between *CDK1* expression and various pathological stages of KIRC, BRCA, KIRP, LUAD ACC, and KICH based on the TCGA database. Figure S5: (A) The mRNA level of *CDK1* was significantly decreased in LAML when compared to normal tissues. (B) C6 enrichment analysis showed that high expression of *CDK1* was also associated with the genes that were upregulated with overexpression of oncogenes, such as E2F1 and KRAS in LAML of TCGA cohort. Figure S6: Kaplan–Meier curves of overall survival analysis in ACC for cancer patients with low *CDK1* expression and high *CDK1* expression. Table S1: Tumor abbreviations of TCGA database. Table S2: The interacted and correlated genes of *CDK1*. Table S3: Raw data of correlation between *CDK1* expression and ESTIMATEScore, ImmuneScore, and StromalScore in tumors of TCGA database.
