# Supplementary material for: An Integrative Human Pan-Cancer Analysis of Cyclin-Dependent Kinase 1 (CDK1)
Source: Cancers (Basel). 2022 May 27;14(11):2658. doi: 10.3390/cancers14112658 (PMC9179585; doi:10.3390/cancers14112658)
Supplement: Supplementary file 1 [file cancers-14-02658-s001.zip › cancers-1689531-supplementary.pdf]

Supplementary material

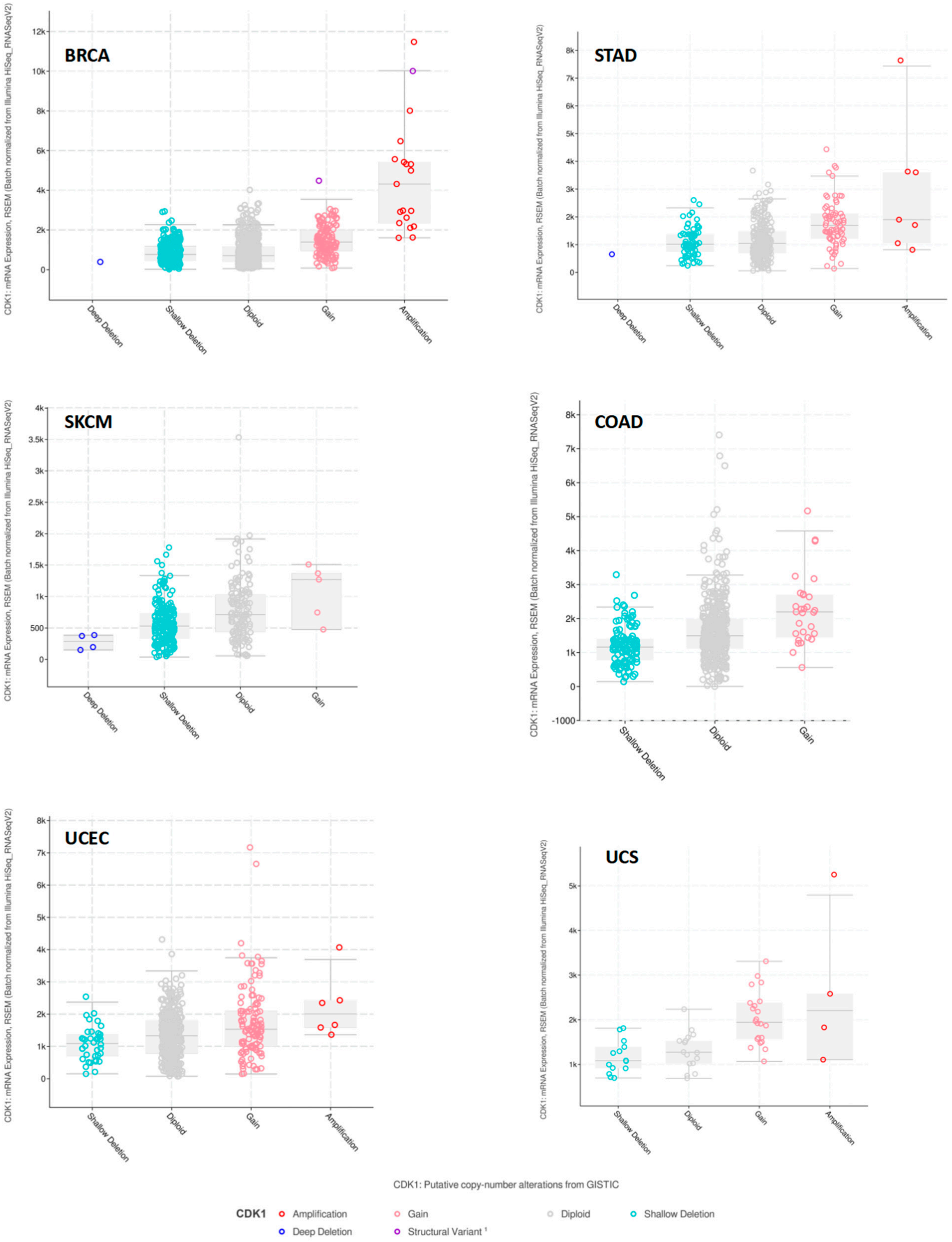

Figure S1. The transcriptional expression of *CDK1* in different types of structural variants in various tumors.

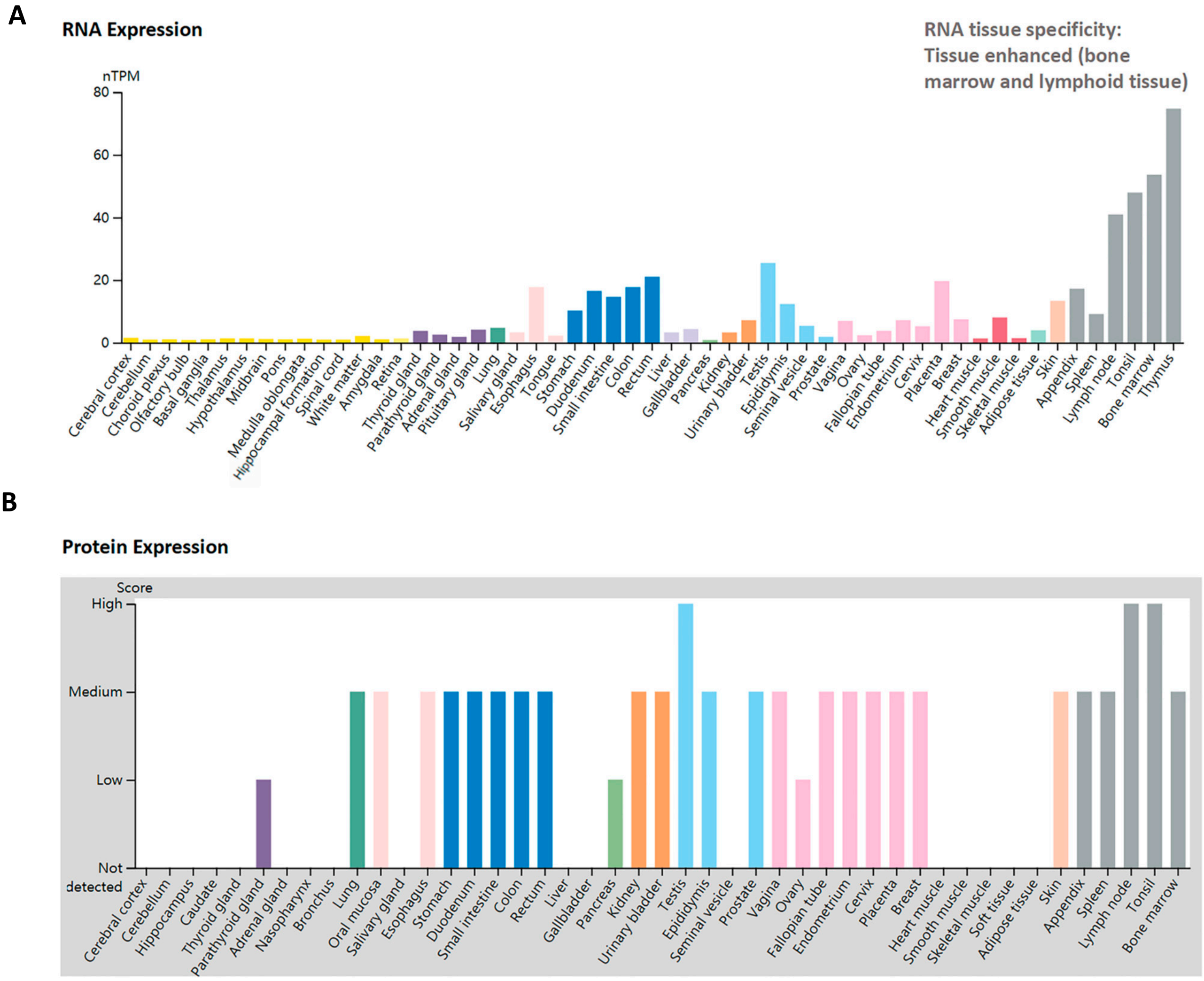

Figure S2. RNA and protein expression of CDK1 in different normal tissues based on the consensus datasets of the HPA and GTEx databases. nTPM, normalized transcripts per million.

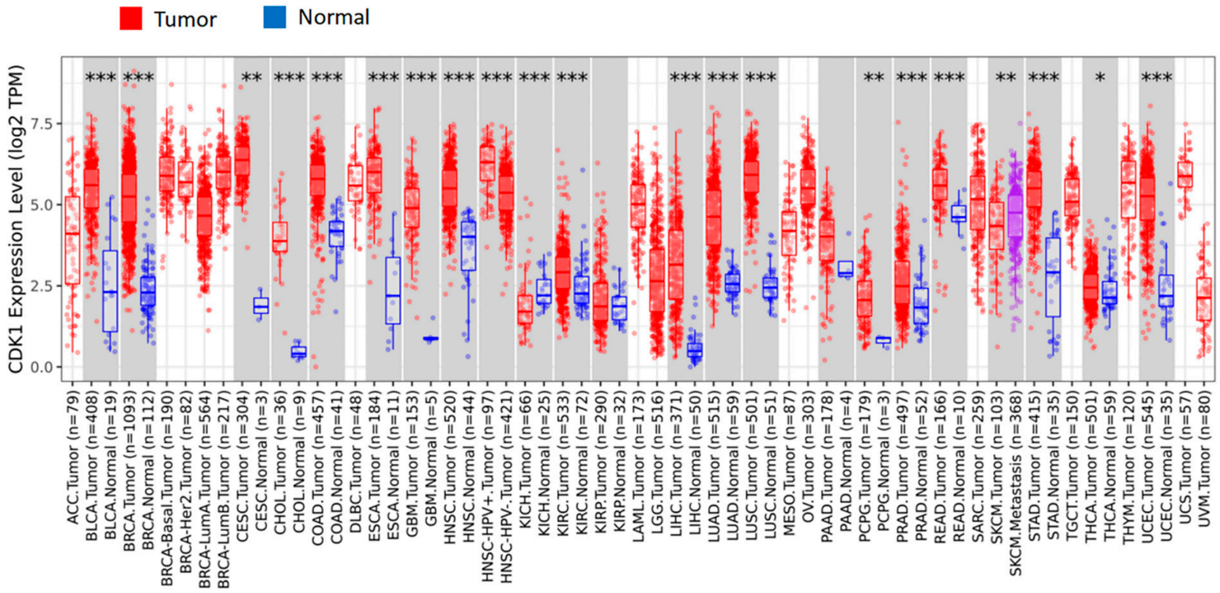

Figure S3. The gene expression of *CDK1* in different cancers based on the TCGA database. *$p < 0.05$, **$p < 0.01$, ***$p < 0.001$.

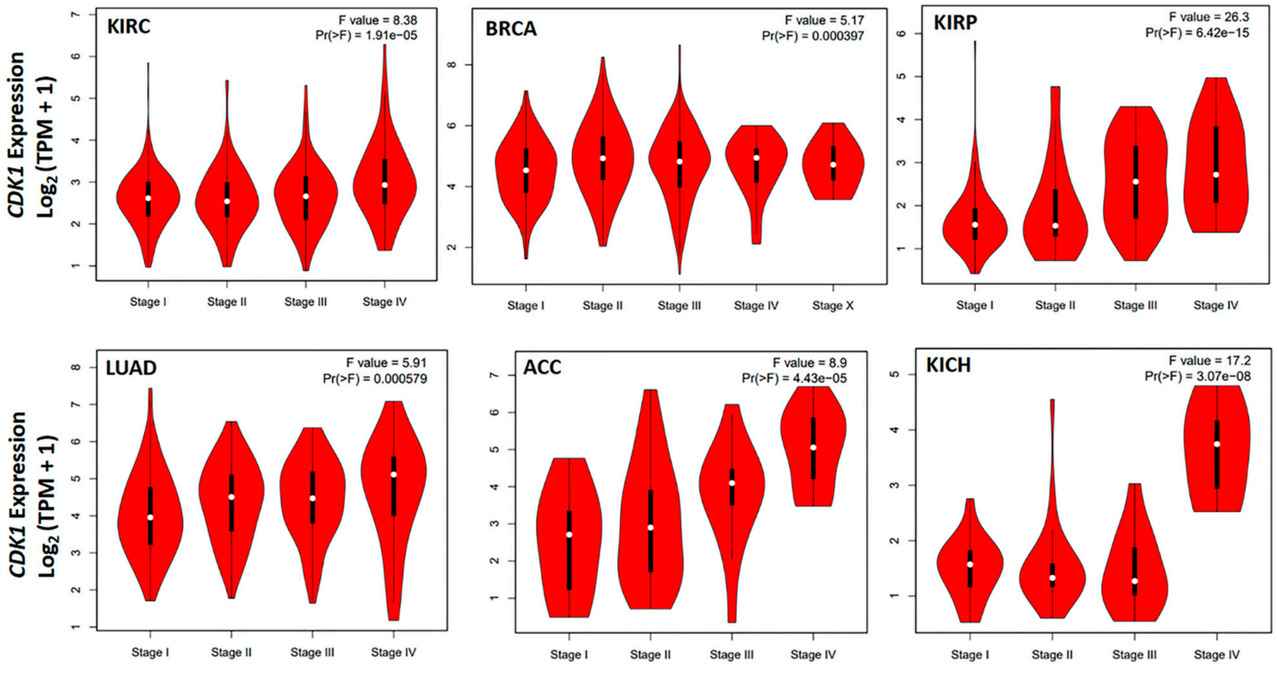

Figure S4. The correlation between *CDK1* expression and various pathological stages of KIRC, BRCA, KIRP, LUAD ACC, and KICH based on the TCGA database.

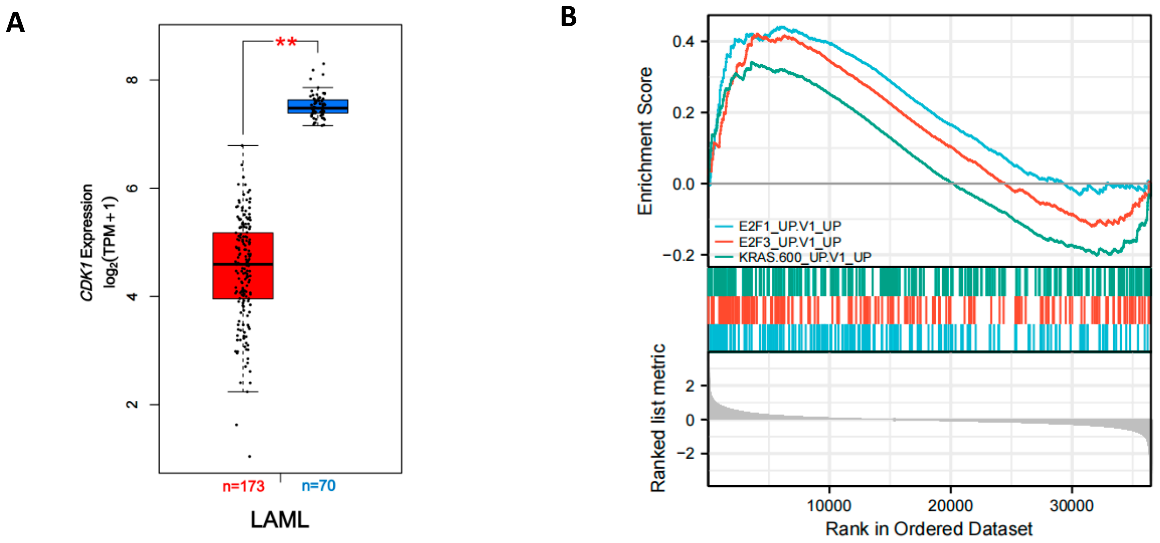

Figure S5. (A) The mRNA expression level of *CDK1* was significantly decreased in LAML when compared to normal tissues. (B) C6 enrichment analysis showed that high expression of *CDK1* was also associated with the genes that were up-regulated with over-expression of oncogenes, such as E2F1 and KRAS in LAML of TCGA cohort. Log2 (TPM + 1) transformed the expression data for plotting. ** $p < 0.01$, in Wilcoxon test. TPM: transcripts per million.

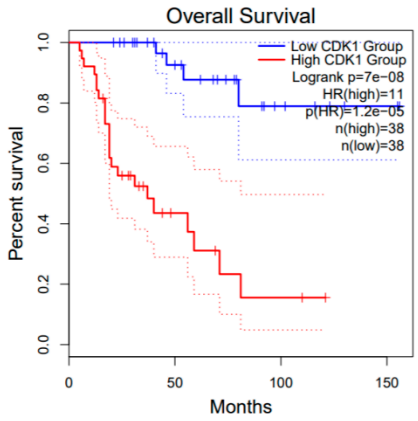

Figure S6. Kaplan–Meier curves of Overall Survival analysis in ACC for cancer patients with low *CDK1* expression and high *CDK1* expression.

Table S1. Tumor abbreviations of TCGA database.

Abbreviations	Full name
LAML	Acute Myeloid Leukemia
ACC	Adrenocortical carcinoma
BLCA	Bladder Urothelial Carcinoma
LGG	Brain Lower Grade Glioma
BRCA	Breast invasive carcinoma
CESC	Cervical squamous cell carcinoma and endocervical adenocarcinoma
CHOL	Cholangiocarcinoma
COAD	Colon adenocarcinoma
ESCA	Esophageal carcinoma
GBM	Glioblastoma multiforme
HNSC	Head and Neck squamous cell carcinoma
KICH	Kidney Chromophobe
KIRC	Kidney renal clear cell carcinoma
KIRP	Kidney renal papillary cell carcinoma
LIHC	Liver hepatocellular carcinoma
LUAD	Lung adenocarcinoma
LUSC	Lung squamous cell carcinoma
DLBC	Lymphoid Neoplasm Diffuse Large B-cell Lymphoma
MESO	Mesothelioma
OV	Ovarian serous cystadenocarcinoma
PAAD	Pancreatic adenocarcinoma
PCPG	Pheochromocytoma and Paraganglioma
PRAD	Prostate adenocarcinoma
READ	Rectum adenocarcinoma
SARC	Sarcoma
SKCM	Skin Cutaneous Melanoma
STAD	Stomach adenocarcinoma
TGCT	Testicular Germ Cell Tumors
THYM	Thymoma
THCA	Thyroid carcinoma
UCS	Uterine Carcinosarcoma
UCEC	Uterine Corpus Endometrial Carcinoma
UVM	Uveal Melanoma

Table S2. The interacted and correlated genes of *CDK1*.

Interacted Genes		Correlated Genes			
BCL2	EZH2	ZWINT	KIF20B	HMMR	CDCA3
CCNA1	FOXM1	KIF11	ASF1B	SGOL1	FANCI
CCNA2	FZR1	CCNA2	LIN9	GINS1	AURKA
CCNB1	GADD45A	CEP55	MCM10	RACGAP1	CENPL
CCNB2	GADD45B	KIF4A	CENPE	BUB1	SASS6
CCNB3	GADD45G	NUF2	KIF14	KIF20A	CENPU
CCND1	GMNN	MAD2L1	ECT2	PRC1	MCM6
CCND3	H1F0	NUSAP1	PARPBP	CDKN3	RAD51AP1
CCNE1	HIST1H1A	CCNB2	OIP5	CENPF	TMPO
CDC20	HIST1H1B	NCAPH	TROAP	KIF18A	ZWILCH
CDC25B	KIF11	KIF2C	RRM2	SPC25	EZH2
CDC25C	LYN	TTK	UBE2C	MTFR2	CDCA8
CDC37	MAPT	UBE2T	LMNB1	CENPI	STIL
CDC6	PKMYT1	TPX2	FAM72D	PLK1	CENPA
CDK2	RB1	NEK2	EXO1	DNAJC9	KPNA2
CDK7	RGCC	KIF23	MASTL	SKA1	FEN1
CDKN1A	RPA1	CCNB1	LRR1	DLGAP5	CDC20
CDKN1B	RPA2	MELK	CKS2	PCNA	PBK
CDKN1C	SAMHD1	NCAPG	CKS1B	CDCA5	DSCC1
CDKN3	SKP2	KIFC1	HJURP	KIAA0101	ORC6
CDT1	TEN1-CDK3	CDC25C	KIF18B	CKAP2L	POLQ
CKS1B	TP53	PLK4	AURKB	BIRC5	CENPK
CKS2	TP53BP1	ASPM	GTSE1	SGOL2	DNA2
E2F1	UBE3A	MKI67	CHEK1	KIF15	TIMELESS
EGFR	WEE1	DEPDC1	FBXO5	NDC80	MCM2

Table S3. Raw data of correlation between *CDK1* expression and ESTIMATEScore, ImmuneScore , and StromalScore in tumors of TCGA database.

**Immu**	**ESTIMATEScore**	**ESTIMATEScore**	**ImmuneScore**	**ImmuneScore**	**StromalScore**	**StromalScore**
Method	pearson_R	pearson_P	pearson_R	pearson_P	pearson_R	pearson_P
TCGA-GBM(N=152)	-0.329996901	3.30E-05	-0.324924087	4.42E-05	-0.308308356	0.000111423
TCGA-GBMLGG(N=656)	0.222333501	8.62E-09	0.200459913	2.25E-07	0.240431393	4.43E-10
TCGA-LGG(N=504)	0.01476449	0.74090359	0.017928469	0.68803289	0.008605225	0.847185544
TCGA-UCEC(N=178)	-0.368607742	4.14E-07	-0.345952825	2.25E-06	-0.321416441	1.22E-05
TARGET-LAML(N=142)	-0.013456803	0.873711287	0.000792102	0.992535459	-0.03173572	0.707715012
TCGA-BRCA(N=1077)	-0.184887517	9.76E-10	-0.064149353	0.0352951	-0.287978711	5.17E-22
TCGA-CESC(N=291)	-0.314079379	4.40E-08	-0.275785029	1.78E-06	-0.284439384	8.07E-07
TCGA-LUAD(N=500)	-0.139452322	0.001773642	-0.116602471	0.009062375	-0.144432988	0.001201462
TCGA-ESCA(N=181)	-0.319259383	1.18E-05	-0.353552258	1.05E-06	-0.23289454	0.001604608
TCGA-STES(N=569)	-0.458887988	5.59E-31	-0.387326404	8.31E-22	-0.463273384	1.29E-31
TCGA-SARC(N=258)	-0.245719417	6.64E-05	-0.219800522	0.000375153	-0.243145836	7.95E-05
TCGA-KIRP(N=285)	-0.018376733	0.757399413	-0.04921266	0.407865521	0.031072389	0.601403631
TCGA-KIPAN(N=878)	0.414122658	1.06E-37	0.349072561	1.47E-26	0.440495874	5.63E-43
TCGA-COAD(N=282)	-0.247019738	2.73E-05	-0.177603158	0.002762069	-0.283204484	1.34E-06
TCGA-COADREAD(N=373)	-0.230898339	6.62E-06	-0.167291395	0.001182898	-0.262363179	2.74E-07
TCGA-PRAD(N=495)	0.002674456	0.952671295	0.011864661	0.792308616	-0.009178552	0.838589577
TCGA-STAD(N=388)	-0.433245573	3.48E-19	-0.3165856	1.76E-10	-0.478487909	1.35E-23
TCGA-HNSC(N=517)	-0.212023688	1.15E-06	-0.085923078	0.05087047	-0.302290334	2.19E-12
TCGA-KIRC(N=528)	0.286006018	2.13E-11	0.251348812	4.74E-09	0.259117192	1.51E-09
TCGA-LUSC(N=491)	-0.326329065	1.20E-13	-0.2698131	1.23E-09	-0.351172983	1.07E-15
TCGA-THYM(N=118)	0.09495536	0.306396676	0.347895703	0.0001133	-0.315203618	0.000507815
TCGA-LIHC(N=363)	-0.072688929	0.166983293	0.024752765	0.638320019	-0.190284366	0.000266075
TARGET-WT(N=80)	-0.248020891	0.026538774	-0.222282493	0.047509231	-0.22848825	0.041495085
TCGA-SKCM-P(N=101)	-0.394972029	4.36E-05	-0.321873169	0.001029528	-0.433391984	5.98E-06
TCGA-SKCM(N=452)	-0.164107241	0.000459903	-0.141530456	0.002562673	-0.172046972	0.000237775
TCGA-BLCA(N=405)	-0.128428606	0.009672849	-0.094140214	0.058373134	-0.146742853	0.003075842
TCGA-SKCM-M(N=351)	-0.165484452	0.001866028	-0.15045272	0.004731425	-0.159626814	0.002706953
TCGA-THCA(N=503)	0.139612726	0.001696515	0.105923715	0.017482519	0.169913701	0.000128579
TARGET-NB(N=153)	-0.437968098	1.50E-08	-0.33263782	2.66E-05	-0.498630212	5.40E-11
TCGA-MESO(N=85)	-0.006633558	0.951953736	-0.076760292	0.485022278	0.099404618	0.365391066
TCGA-READ(N=91)	-0.187421198	0.075244882	-0.138617786	0.190065364	-0.208328327	0.047518078
TCGA-OV(N=417)	-0.143340741	0.003351886	-0.106302479	0.029976933	-0.161096339	0.000962058
TCGA-UVM(N=79)	-0.016433489	0.885701379	0.023122966	0.839703633	-0.102265609	0.369813438
TCGA-PAAD(N=177)	0.026274447	0.728484613	0.030489743	0.687051738	0.019193661	0.799828413
TCGA-TGCT(N=132)	-0.501952104	8.70E-10	-0.586596172	1.46E-13	-0.081421146	0.353354391
TCGA-UCS(N=56)	-0.085100461	0.532888338	-0.167774553	0.216467614	0.033138258	0.808425731
TCGA-LAML(N=214)	0.117640557	0.086012053	-0.063608979	0.354442158	0.325589727	1.13E-06
TARGET-ALL(N=86)	0.04908078	0.653599938	0.007054411	0.948601238	0.1175423	0.281108149
TCGA-PCPG(N=177)	-0.042875877	0.570955036	-0.129664933	0.085409446	0.045801181	0.544949428
TCGA-ACC(N=77)	-0.302020842	0.007595904	-0.315686407	0.005162315	-0.253596973	0.02605293
TARGET-ALL-R(N=99)	0.04543292	0.655206219	-0.001739293	0.986368034	0.13032068	0.198548802
TCGA-DLBC(N=46)	0.059620583	0.693884849	-0.073526008	0.627242321	0.184907234	0.218610997
TCGA-KICH(N=65)	0.024789366	0.844601995	-0.033018591	0.794007415	0.097556043	0.439458693
TCGA-CHOL(N=36)	-0.302736501	0.07270252	-0.263844535	0.119970922	-0.324108899	0.053800526
